# THE FOXO3 LONGEVITY GENOTYPE ATTENUATES IMPACT OF HYPERTENSION ON CEREBRAL MICROINFARCT RISK

**DOI:** 10.1093/geroni/igad104.1940

**Published:** 2023-12-21

**Authors:** Richard Allsopp, Kazuma Kakagawa, Kamal Masaki, Brian Morris, D Craig Wilcox, Bradley Willcox

**Affiliations:** University of Hawaii, Honolulu, Hawaii, United States; Queen’s Hospital, Honolulu, Hawaii, United States; Kuakini Medical Center, Honolulu, Hawaii, United States; Kuakini Medical Center, Honolulu, Hawaii, United States; Okinawa International University, Ginowan, Okinawa, Japan; Kuakini Medical Center, Honolulu, Hawaii, United States

## Abstract

The G allele of the FOXO3 SNP rs2802292 is associated with human resilience and longevity and has recently been shown to attenuate the impact of hypertension on the risk of intracerebral hemorrhage. We now determined whether FOXO3 G-allele similarly attenuated the impact of hypertension on the risk of cerebral microinfarct (CMI). From a prospective population-based cohort of American men of Japanese ancestry (Kuakini Honolulu Heart Program), age-adjusted prevalence of any (1 or more) CMI on brain autopsies was assessed. Logistic regression models, adjusted for age of death, cardiovascular risk factors, FOXO3 and APOE-ε4 genotypes, were utilized to determine the predictors of any CMI. A potential interaction effect of FOXO3 and hypertension was also analyzed. Among 809 men with complete data, 511 (63.2%) had evidence of any CMI. A full multivariable model demonstrated that body mass index (OR: 1.07, 95% CI: 1.01–1.14; p=0.012) was the only significant predictor of CMI, while hypertension was a borderline predictor (OR: 1.44, 95% CI: 1.00–2.08; p=0.052). A significant interaction between FOXO3 G-allele genotype and hypertension was observed (p=0.02). In the stratified analyses, among the participants without the longevity-associated FOXO3 G-allele, hypertension was a strong predictor of CMI (OR: 2.25, 95% CI: 1.34–3.77;, p=0.0021), while among those with the longevity-associated FOXO3 G-allele, hypertension was not (OR: 0.88, 95% CI: 0.51–1.54; p=0.66). These results suggest that the longevity-associated FOXO3 G-allele attenuated the impact of hypertension on the risk of CMI. More studies of other populations is needed to further validate this interesting relation.

